# Patient-Reported Outcomes and Provocative Testing in Peripheral Nerve Injury and Recovery

**DOI:** 10.1055/s-0043-1764352

**Published:** 2023-04-21

**Authors:** Albin John, Stephen Rossettie, John Rafael, Cameron T. Cox, Ivica Ducic, Brendan J. Mackay

**Affiliations:** 1 Texas Tech University Health Sciences Center, Lubbock, Texas; 2 Washington Nerve Institute, McLean, Virginia

**Keywords:** provocative testing, functional assessment, nerve recovery, peripheral nerve injury, patient-reported outcomes

## Abstract

**Background** Peripheral nerve function is often difficult to assess given the highly variable presentation and subjective patient experience of nerve injury. If nerve assessment is incomplete or inaccurate, inappropriate diagnosis and subsequent treatment may result in permanent dysfunction.

**Objective** As our understanding of nerve repair and generation evolves, so have tools for evaluating peripheral nerve function, recovery, and nerve-related impact on the quality of life. Provocative testing is often used in the clinic to identify peripheral nerve dysfunction. Patient-reported outcome forms provide insights regarding the effect of nerve dysfunction on daily activities and quality of life.

**Methods** We performed a review of the literature using a comprehensive combination of keywords and search algorithms to determine the clinical utility of different provocative tests and patient-reported outcomes measures in a variety of contexts, both pre- and postoperatively.

**Results** This review may serve as a valuable resource for surgeons determining the appropriate provocative testing tools and patient-reported outcomes forms to monitor nerve function both pre- and postoperatively.

**Conclusion** As treatments for peripheral nerve injury and dysfunction continue to improve, identifying the most appropriate measures of success may ultimately lead to improved patient outcomes.

## Introduction

The incidence of peripheral nerve injury (PNI) is estimated at 16.9 per 100,000 citizens in the United States. 1 Symptoms of PNI present along a broad spectrum depending on the severity and mechanism of injury. 1 In neurotmetic injuries, both the nerve and its entire surrounding sheath are disrupted. Axonometric injuries involve the axons and myelin sheath but spare the endoneurium, perineurium, and epineurium. 2 After the initial injury, Wallerian degeneration commences and is necessary for eventual regeneration. 3 This degenerative process is known to incite inflammatory processes. 4 The combination of disrupted blood flow and inflammation can lead to edema, elevated intraneural pressure, and potential damage to the myelin sheath. 5 A process known as the “cumulative injury cycle” can occur when elevated pressure triggers further blood flow restriction and inflammation. 5

Nerve pain or disability caused by nerve inflammation often develops after nerve surgery or traumatic injury. 6 In traumatic nerve injuries, surrounding tissues are often involved and multisystem involvement has been linked to suboptimal outcomes. 1 7 While treatment decisions for severe nerve injuries (e.g., complete transection) are relatively straightforward, incomplete transections, or nontrauma nerve deficiency are often difficult to diagnose, treat, and monitor due to their more subtle presentation.

A timely, accurate assessment of nerve function is critical as the delayed diagnosis can negatively affect final outcomes. 8 9 If significant PNI is left untreated, complete functional recovery is unlikely. 10 Even with prompt diagnosis and treatment, PNIs still challenge surgeons, and treatment algorithms are continually improving. 11 12 13 As our understanding of nerve repair and generation evolves, so have tools for evaluating the function of peripheral nerves and nerve-related impact on quality of life.

Given the complexity and variability of PNIs, it is unlikely that a single assessment modality will provide a complete picture of a given patient's nerve injury or recovery status. Complex cases require careful consideration when choosing between assessment tools, 14 and surgeons may benefit from a detailed, comprehensive view of the literature evaluating individual tools within broader categories such as motor assessments, sensory assessments, pain assessments, provocative testing, and patient-reported outcomes forms. 15 We performed a review of the literature on provocative testing and patient-reported outcomes forms, with special attention to the advantages, disadvantages, recent improvements, and potential role in nerve assessment algorithms.

## Methods

### Development Process

The authors performed a systematic review across multiple databases using a comprehensive combination of keywords and search algorithms according to the Preferred Reporting Items for Systematic Reviews and Meta-Analyses (PRISMA) guidelines. 16 The literature search focused on clinical data regarding the assessment of sensory and pain recovery after PNI was undertaken to define the utility of each assessment tool.

### Literature Search

A systematic literature review was conducted to identify study abstracts for screening. The databases used included PubMed/MEDLINE, EMBASE, Cochrane, and Google Scholar databases using the controlled terms: “humans” and “peripheral nerve injuries” and “patient-related outcomes” or “function” or “assessment” or “recovery” or “outcome”. Manual additions to our search query were made using the key terms: “provocative test,” “functional assessment,” “nerve assessment,” “nerve recovery assessment,” “nerve injury testing,” “nerve testing,” “peripheral nerve assessment,” and “nerve function testing”. Search dates were from January 1960 to December 2020. After the assessment of eligibility, three authors extracted data from the marked articles. Important parameters that were recorded when available included: the year of the study, number of patients in the study, sensitivity and specificity of the tools assessed, benefits and limitations of tools assessed, opportunities for improvement, and clinical roles in nerve recovery assessment.

### Study Eligibility

**Fig. 1:**
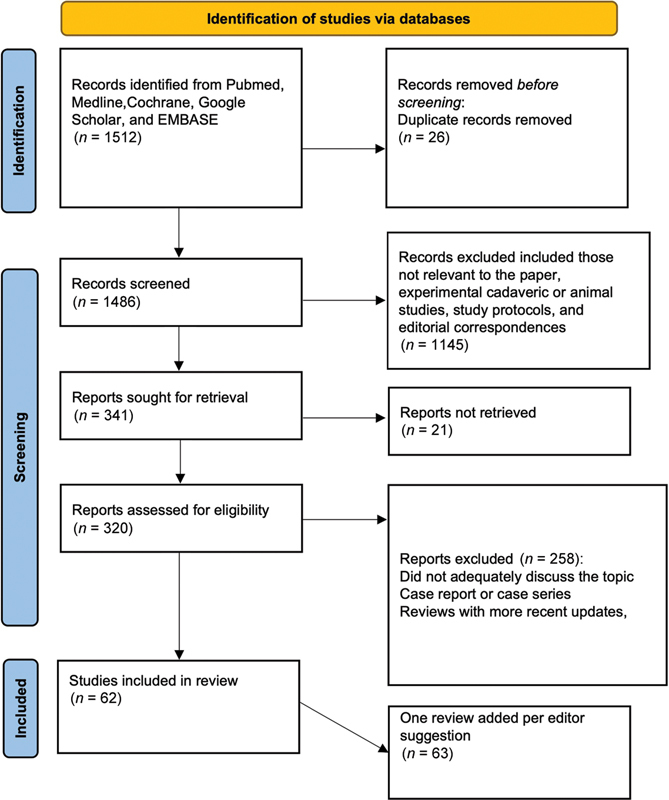
PRISMA guideline flow diagram

A minimum of two reviewers worked independently to further review and screen abstracts and titles. All articles that reported the pathogenesis of sensory deficits secondary to nerve damage and those that assessed various tools used to measure sensory recovery and pain assessment tools were included. Only articles in English were reviewed. Full texts of articles were assessed during screening if there was uncertainty on whether the article should be included. Article titles and abstracts that did not address our research question objective were excluded. Further full-text assessment of the selected articles was done during which articles that did not address provocative testing and patient-reported outcome assessments were removed. The PRISMA diagram in Fig. 1 further describes the literature evaluation process.

### Data Extraction

After the assessment of eligibility, three authors extracted data from the marked articles. Important parameters that were recorded when available included: the year of the study, number of patients in the study, sensitivity and specificity of the tools assessed, benefits and limitations of tools assessed, opportunities for improvement, and clinical roles in nerve recovery assessment.

### Ethical Consideration

As this review is a narrative review, ethical review or approval was not required. No patient information or identifying features were included in this study.

## Results

### Provocative Testing

Provocation tests have been used for over a century to screen for neurologic compromise. 17 As the name suggests, these are designed to agitate the nerve in question with certain responses expected for impaired versus normal nerves. A summary of these tests is provided in Table 1 .

**Table 1:** Provocative Testing

Test	Description	Areas of use	Positive result	Additional information	Source
Tinel's sign	Performed by tapping firmly over the course of a nerve to determine the site of compression or regeneration	Wrist (median nerve), Ankle (Sural and Peroneal nerves)	“Pins and needles” sensation upon proximal tapping of nerve	Used for initial screening of compressive neuropathy including carpal tunnel, Guyon canal, tarsal tunnel, and cubital tunnel syndromes. Lower sensitivity than Phalen's and Durkan's test.	17, 18, 19
Phalen's test	Performed by having patients passively drop wrists in complete flexion for 60 seconds.	Wrist (median nerve)	Numbness or paresthesia in the median nerve distribution after 60 seconds	Used for initial screening of compressive neuropathy but relies on adequate ROM. More sensitive and specific than Tinel Sign, especially in the mild to moderate stage of nerve compression. Less sensitive than Durkan's test.	20–22
Durkan's test	Performed by physician pressing on the edge of the carpal ligament at the proximal wrist crease with the patient's wrist in a neutral position.	Wrist (median nerve)	Increase in paresthesia in the median nerve distribution	Used for initial screening of compressive neuropathy. More sensitive but less specific than both Tinel's and Phalen's tests.	23, 24
Scratch Collapse Test (SCT)	Performed by lightly agitating the patient's skin over the area of suspected nerve compression, followed immediately by bilateral resisted shoulder external rotation.	Wrist (median nerve) and Elbow (ulnar nerve)	Temporary loss of muscle resistance in the affected arm	SCT is not a standalone screening tool, but literature indicates it may provide ancillary benefit in complex presentations of compressive neuropathy. Literature has been controversial, but the seminal study indicated diagnostic accuracy of 82 and 89% for carpal and cubital tunnel syndrome, respectively.	25

Abbreviation: ROM, range of motion.

### Tinel's Sign—Hoffman and Tinel (1915)

#### Aims/Advantages

Tinel's test is performed by tapping firmly over the course of a nerve to determine the site of compression or regeneration. ^18,^ 19 A positive result is defined as a “pins and needle” sensation elicited by proximal tapping. It is easy to perform and frequently used in clinics as an initial screening tool (for compressive neuropathy) or to track the progress of nerve regeneration. 18 While reports vary regarding sensitivity (23–67%), Tinel's test has demonstrated high specificity (95–99%). 19 In the context of chronic compression, it is most commonly used to diagnose carpal tunnel syndrome but is also used in other neuropathies, including tarsal tunnel, cubital tunnel, and Guyon's canal syndromes. 19 Following traumatic nerve injury and/or repair, Tinel's sign is used to track regeneration as the site at which a tingling sensation is elicited will migrate distally with the regenerating nerve fibers. 18

#### Disadvantages/Criticisms

The absence of Tinel's sign does not necessarily rule out compression and/or axonal loss, particularly in mild or moderate presentations. 20 In carpal tunnel syndrome, Phalen's test and Durkan's test (described in later sections) have demonstrated superior sensitivity and Phalen's test has demonstrated greater specificity. 19

#### Role in Nerve Assessment Algorithm

Tinel's test may be used for initial screening, particularly in compressive neuropathy, as well as tracking the progression of nerve regeneration following injury and/or repair. While more quantitative clinical tests are often needed to determine the precise location and extent of neurologic compromise, this is an accessible adjunct for initial evaluation.

### Phalen's Test—Phalen (1951)

#### Aims/Advantages

Phalen's test is performed by having patients passively drop wrists in complete flexion for 60 seconds. A positive result is defined as resulting numbness or paresthesia in the median nerve distribution. ^21,^ 22 The reverse Phalen's is performed in a similar manner with active extension. 23 Phalen's test has shown higher sensitivity and specificity when directly compared to Tinel's test and is more likely to show a positive result in the mild-to-moderate stages of nerve compression. 20 22

#### Disadvantages/Criticisms

Phalen's test may not be adequate to test patients with a limited range of motion. 20 Despite improvements in sensitivity compared to Tinel's test, Phalen's test is considered less sensitive than Durkan's test (described in the following section). 20

#### Role in Nerve Assessment Algorithm

Phalen's test may be used for initial screening in suspected compressive neuropathy. While additional tests are often necessary to determine the extent of neurologic compromise, this test provides valuable data and is an accessible component of the initial exam.

### Durkan's Test—Durkan (1991)

#### Aims/Advantages

The Durkan test was developed with the understanding that direct pressure will increase neurologic dysfunction of an impaired median nerve, presumably via amplification of ischemic conditions. ^23,^ 24 To perform this test, the physician presses on the edge of the carpal ligament at the proximal wrist crease with the patient's wrist in a neutral position. Increase in paresthesia in the median nerve distribution is taken as a positive result. 20 Durkan's test has shown higher sensitivity compared to Tinel's and Phalen's tests. 25

#### Disadvantages/Criticisms

While Durkan's test has high sensitivity, reports indicate that both Tinel's and Phalen's tests have greater specificity. 25

#### Role in Nerve Assessment Algorithm

Durkan's test is a valuable tool for initial screening, particularly for compressive neuropathy. While further clinical tests (e.g., electromyography (EMG), Tinel's sign, Carpal Tunnel Syndrome [CTS-6], Grip/Pinch strength, Semmes Weinstein monofilament (SWM), and/or two point discrimination (2-PD)) may be needed to confirm neurologic compromise in more complex cases, Durkan's test is a valuable part of the initial clinical exam.

### Scratch Collapse Test—Cheng et al (2008)

#### Aims/Advantages

The scratch collapse test (SCT), developed by Susan MacKinnon, is a diagnostic physical exam for compressive neuropathy (e.g. carpal and cubital tunnel syndrome). 26 The test is performed by lightly agitating the patient's skin over the area of suspected nerve compression, followed immediately by bilateral resisted shoulder external rotation. 26 Temporary loss of muscle resistance in the affected arm is considered a positive result. 26 The seminal study on SCT showed a diagnostic accuracy of 82 and 89% for carpal and cubital tunnel syndrome, respectively, as well as improved sensitivity (compared to Tinel's sign and elbow flexion/compression tests) for both carpal and cubital tunnel. 26

#### Disadvantages/Criticisms

Subsequent studies have called the sensitivity and interrater reliability of the SCT into question. 27 28 Multiple reports have challenged the proposed mechanism of action utilized by SCT—the cutaneous silent period (CuSP). 28 The CuSP is described as a transient decrease in EMG activity observed after a noxious stimulus of a nerve. 28 Some studies have shown that the CuSP was prolonged in moderate CTS patients, but absent in severe CTS patients. Others have shown that CuSP duration in CTS patients did not differ significantly from healthy controls. 28

#### Improvements

The creator of the SCT later published a comprehensive guide to improve interrater reliability and specificity, replete with video demonstrations of various steps, including positioning, establishing a baseline of balanced bilateral external rotation, appropriate cutaneous irritation (not always scratch as the name implies), using ethyl chloride to create a false-negative baseline (i.e., response without any cutaneous irritation), and interpretation of results. 29 The report also includes guidelines to determine which clinical scenarios might benefit from the use of SCT. 29

#### Role in Nerve Assessment Algorithm

Careful use of the SCT per the updated guidelines of its creator 29 may provide useful data in cases where the diagnosis remains unclear despite a battery of gold-standard clinical tests (e.g. EMG, Tinel's sign, CTS-6, Grip/Pinch strength, SWM, and/or 2-PD). While the literature indicates that the SCT is not a standalone screening tool, it may provide ancillary benefits in complex presentations of compressive neuropathy. 28 30 31

## Patient-Reported Outcome Measures

Patient-reported outcomes measures (PROMs) give physicians an individualized view of a patient's recovery. These may also help determine whether patients are limited by pain management rather than physical impairment as they progress along the trajectory of recovery. 32 33 These forms range from broad to injury-specific, with the value of each depending on the clinical context for use ( Table 2 ).

**Table 2:** Patient-reported outcomes measures

PROM	Description	Normal values	Additional Information	Source
Short Form-36	Measures a patient's physical and mental health	Physical Component: 50 ± 10.0, Mental Component: 50 ± 10.0	8 domains of daily activity are assessed. Highly generalizable and can be used across various pathologies of peripheral nerve injury and recovery.	38
Patient Specific Functional Scale	Self-reported questionnaire that identifies specific tasks that patients are unable to complete as a result of their injury.	Increase in 3 or more demonstrates clinically significant functional change	11-point scale that assesses level of difficulty performing tasks and is repeated throughout recovery. Good test-retest reliability and validity.	41, 42
Disabilities of the Arm, Shoulder, and Hand	Measures upper extremity disability	Normal = 10.1 ± 14.68, lower scores indicate less disability	30-item tool assessing functional status of upper extremity as a single unit. Scored on a scale of 1 to 5 with higher scores indicating higher level of disability. Useful for CTS and predicting level of disability secondary to traumatic peripheral nerve injury.	44, 51
Michigan Hand Outcomes Questionnaire	Measures recovery of hand function after carpal tunnel release, rheumatoid arthritis, Dupuytren contracture, amputation, etc.	Minimal clinical important difference = 3.0 to 23.0 depending on pathology	67-item questionnaire graded on a 1–4 Likert Scale to assess impact of hand impairment on daily activities.	55
Boston Questionnaire for Carpal Tunnel Syndrome (CTS)	Hand function and symptom severity in patients with Carpal Tunnel Syndrome	Higher scores indicate decreased functional status	11-item symptom specific scale and an 8-item function-specific scale scored on a 1–5 Likert Scale.	40,46,56
6-item CTS Symptom Scale	Measures carpal tunnel syndrome symptom severity and functional disability	12 or greater indicates median nerve damage	Useful for quick assessment of median nerve function following carpal tunnel release.	58

### Short Form-36—Ware and Sherbourne (1992)

#### Aims / Advantages

The Short Form-36 (SF-36) measures a patient's physical and mental health. 34 35 It is the most commonly used Health-Related Quality of Life (HRQoL) assessment tool. 36 The SF-36 consists of eight domains to address different areas of daily activity impacted by patients' injury and recovery. 15 35 While it highly generalizable and can be used across multiple diseases and injuries, the tool is not specific to any particular injury or disease pattern. The SF-36 has internal consistency and reliability. 35 36 The generic nature of the SF-36 also allows researchers to compare the impact on quality of life across diseases and populations. 37

#### Disadvantages/Criticisms

The SF-36 has some redundancy and can be confusing to responders. Furthermore, scoring varies by patients' interpretations of the questions. 35 38 Another disadvantage of this survey is that the elderly may require reading assistance, which may introduce further variability. 35 The interconnected components of the scales used in the SF-36 can result in difficulty interpreting outcomes. 35

#### Improvements

The initial SF-36 had both floor and ceiling effects because of limited response options. The current version has increased its response options to combat these effects. 35 Despite these changes, floor and ceiling effects persist in the role limitations and emotional functioning subscales. 36

#### Role in Nerve Assessment Algorithm

The SF-36 is recommended to understand patients' overall mental and physical well-being. In the context of nerve assessment, the SF-36 should be used as a supplementary rather than primary measure. 36 When choosing between the quality of life measures, more specific tools such as the DASH or BSQ should be given preference over the SF-36.

### Patient-Specific Functional Scale—Stratford et al (1995)

#### Aims/Advantages

This self-reported questionnaire identifies patient-specific tasks are difficult for individuals to complete as a result of their injury. 39 40 41 Patients are asked to list five activities that are important to them and that they are unable to perform as a result of their injury. The level of current difficulty reported by the patient is graded on an 11-point scale. The patient-specific functional scale (PSFS) is repeated after the intervention to assess functional recovery. 42 PSFS has good test-retest reliability and validity. 41 42 Clinically significant functional change is defined as an increase or decrease of three or more PSFS points. It is an easily administered tool that has a low responder burden. 41 42

#### Disadvantages/Criticisms

While scores are useful for understanding a patient's particular difficulty, the activities chosen by each patient are specific to the individual. As a result, the PSFS is not amenable to comparisons across patients. 40 41 42 The PSFS cannot be used in conditions for which it has not been specifically adapted. 42 The PSFS also has a floor effect as the tool has very little ability to show a decline in function. 41

#### Improvements

Applications of PSFS beyond musculoskeletal disorders, such as neurologic or cardiopulmonary conditions (aside from chronic obstructive lung disease), have yet to be explored. Such adaptations could extend the use of PSFS in practice. 42 Additionally, to avoid the floor effect, patients may be asked to include some activities that they are having only “a little bit” of difficulty with. Using the postinterventional score on these activities, the tool can be used to measure functional deterioration. 41

#### Role in Nerve Assessment Algorithm

This tool is particularly useful in understanding functional change in musculoskeletal disorders as a result of intervention. 42 It can also be used to assess patients who have exceptional recovery; thus, may be used in cases where other tools experience the ceiling effect. 42 While the PSFS is not a standalone assessment modality, it may assist in tailoring the assessment algorithm to individual patients and their respective goals.

### Disabilities of the Arm, Shoulder, and Hand—Hudak et al (1996)

#### Aims/Advantages

The Disabilities of the Arm, Shoulder, and Hand (DASH) score, introduced in 1996, is used to measure disability in the upper extremity. 43 It is a 30-item tool assessing the functional status of the upper extremities as a single unit (both right and left arms). 40 44 Individual items are scored on a scale of 1 to 5, with higher total scores indicating a higher level of disability. This tool has shown utility in assessing outcomes of carpal tunnel release and in predicting levels of disability in traumatic peripheral nerve injuries. 40 The DASH has good reliability and validity. 45

#### Disadvantages/Criticisms

The DASH score is unique in that it assesses the overall ability to complete an activity without separating affected and nonaffected hands or arms. While this may be an advantage in understanding a patient's entire recovery journey, it lacks information on the recovery of the affected limb and may describe coping or overcompensation more than recovery. 46 Furthermore, if respondents do not complete the questionnaire fully (if they skip more than three responses), the questionnaire cannot be used. 47 The DASH questionnaire has redundancy in items as evidenced by an elevated Cronbach alpha (0.97). 44 48 The DASH also has a ceiling effect such that the precision of assessing an individual with higher functional status is diminished. The ceiling effect occurs when performance in a category exceeds the ability of a tool to defect any deficiency; once the ceiling is reached, dysfunction and/or further improvements cannot be measured. 49 Some questions are considered too complex for certain patients. 50

#### Improvements

The QuickDASH form was created in 2005 to reduce the length and burden of the DASH survey. 15 The QuickDASH is based on the patient's perception of pain and functional impairment. Normal populations have a score of 10.1 ± 14.68 (out of 100 points), with lower scores indicating less disability. This survey has high internal consistency and validity. However, if more than one item is skipped, a score cannot be calculated. 44 51

#### Role in Nerve Assessment Algorithm

While the DASH is a more comprehensive questionnaire, the QuickDASH can provide an overall assessment of upper extremity disability similar to the DASH. 44 The DASH is not recommended for certain injuries, such as Dupuytren's contracture, where the ceiling effect may come into play. 50

### Michigan Hand Outcomes Questionnaire—Chung et al (1998)

#### Aims/Advantages

The MHQ was designed to evaluate hand function after carpal tunnel release, rheumatoid arthritis, and even amputation. ^52^ This 67-item questionnaire can assess the impact of hand impairment on daily activities using a 1 to 5 Likert scale with high sensitivity. Given its specialized focus, the MHQ offers more detailed insights than the DASH form. 15 40 53 The MHQ can effectively discern even small functional differences in the affected versus nonaffected hand. 47 53 54 55 It is also unique in that it addresses patient satisfaction with hand aesthetics. 52 53 The MHQ is easy to use and has high test-retest reliability and internal inconsistency. 52

#### Disadvantages/Criticisms

Observers have noted that when respondents are answering the same question twice on the MHQ, once for the injured hand and once for the noninjured hand, they seemed to lose their attention. 47 Furthermore, the entire questionnaire must be completed to calculate a score. 47 Similar to the DASH, the MHQ is subject to the ceiling effect, especially in traumatic conditions that improve rapidly. 46 54 The MHQ also has a high Cronbach's alpha, indicating redundancy. 52

#### Improvements

The Brief Michigan Hand Outcomes Questionnaire (brief MHQ) was developed to reduce respondent burden of the original MHQ. 52 It is half the length of the old questionnaire and has high reliability and validity. The brief MHQ has demonstrated efficacy in assessment of rheumatoid arthritis, carpal tunnel, distal radius fracture, and joint osteoarthritis. 54

#### Role in Nerve Assessment Algorithm

The MHQ (or brief MHQ) is most commonly used for hand outcomes assessment in arthritis and trauma. 54 It may be used in less severe injuries that DASH may not be indicated for, such as Dupuytren contracture. The MHQ can be used to compare the functional status of one hand to the contralateral side. Of note, the MHQ and the brief MHQ are not recommended for patients with large functional deficits as these are better assessed using the DASH. 54

### Boston Questionnaire for Carpal Tunnel Syndrome—Levine et al (1993)

#### Aims/Advantages

The Boston questionnaire (BQ), sometimes referred to as the CTS questionnaire, is comprised of two scales: an 11-item symptom-specific scale and an 8-item function-specific scale. 56 It is used to evaluate hand function and symptom severity in patients with carpal tunnel syndrome. Respondents indicate their ability to accomplish eight tasks on a 1 to 5 Likert scale. Higher total scores indicate decreased functional status. The BQ has high validity, internal consistency, and sensitivity. 40 46 57 It is a comprehensive assessment that can quantify symptom severity as early as 2 weeks postoperatively. 15 The BQ can be completed in under 10 minutes. 57

#### Disadvantages/Criticisms

While this questionnaire is sensitive to clinical changes, results are only weakly correlated with physical examinations such as 2PD and Semmes–Weinstein monofilament testing. 46 Due to its two distinct constructs, functional status and severity of symptoms, the overall score may not reveal specific limitations. 57

#### Improvements

The CTS-6 (described in the following section) was developed as an improvement on the CTS, with 11 total items to increase compliance and eliminate redundancy. 58

#### Role in Nerve Assessment Algorithm

The BQ is most useful for monitoring median nerve status following carpal tunnel release and should be used as an adjunct to corroborate findings of quantitative sensory and motor tests.

### Six-Item Carpal Tunnel Syndrome Symptom Scale—Atroshi et al (2009)

#### Aims/Advantages

The 6-item CTS Symptom Scale (CTS-6), developed by Atroshi and referred to as CTS-6, is a subjective measure of carpal tunnel syndrome symptoms that assesses symptom severity and functional disability. 58 59 The questionnaire was adapted from the BQ and developed as a low-cost, easy-to-use alternative to objective nerve conduction studies following surgical intervention for carpal tunnel disease. Although initially intended for postsurgical assessments, the CTS-6 has also been validated as a measure of poststeroid injection symptoms. 58

The CTS-6 has shown a strong correlation to the QuickDASH and the BQ from which it was derived. This 6-question form is more concise and straightforward without sacrificing valuable data points or increasing error. The CTS-6 is also reliable and responsive in tracking postoperative changes. 58 60 Items removed from the CTS to develop the CTS-6 were ultimately deemed redundant or nonessential to characterize median nerve status. 58

#### Disadvantages/Criticisms

While the CTS-6 has a nonspecific functional ability scale, it cannot be adequately used to understand many upper extremity nerve conditions. Furthermore, if patients skip more than 1 item, a score cannot be calculated. 58

#### Improvements

Researchers, using Rasch Measurement Theory analysis, noted that the summing of scores from the 6 items may not be valid due to the lack of latent unidimensionality. They proposed splitting up the score summation such that questions 1,2, and 5 for pain were grouped together while questions 3,4,6 for numbness were grouped separately. 61

#### Role in Nerve Assessment Algorithm

This questionnaire can be used for a quick assessment of median nerve function following carpal tunnel release. 58 If patients are receiving multiple tests, the use of CTS-6, instead of the Boston Questionnaire, can reduce respondent burden while providing a complete picture of hand status. The test may also be used in conjunction with the QuickDASH. 58

## Discussion

**Fig. 2:**
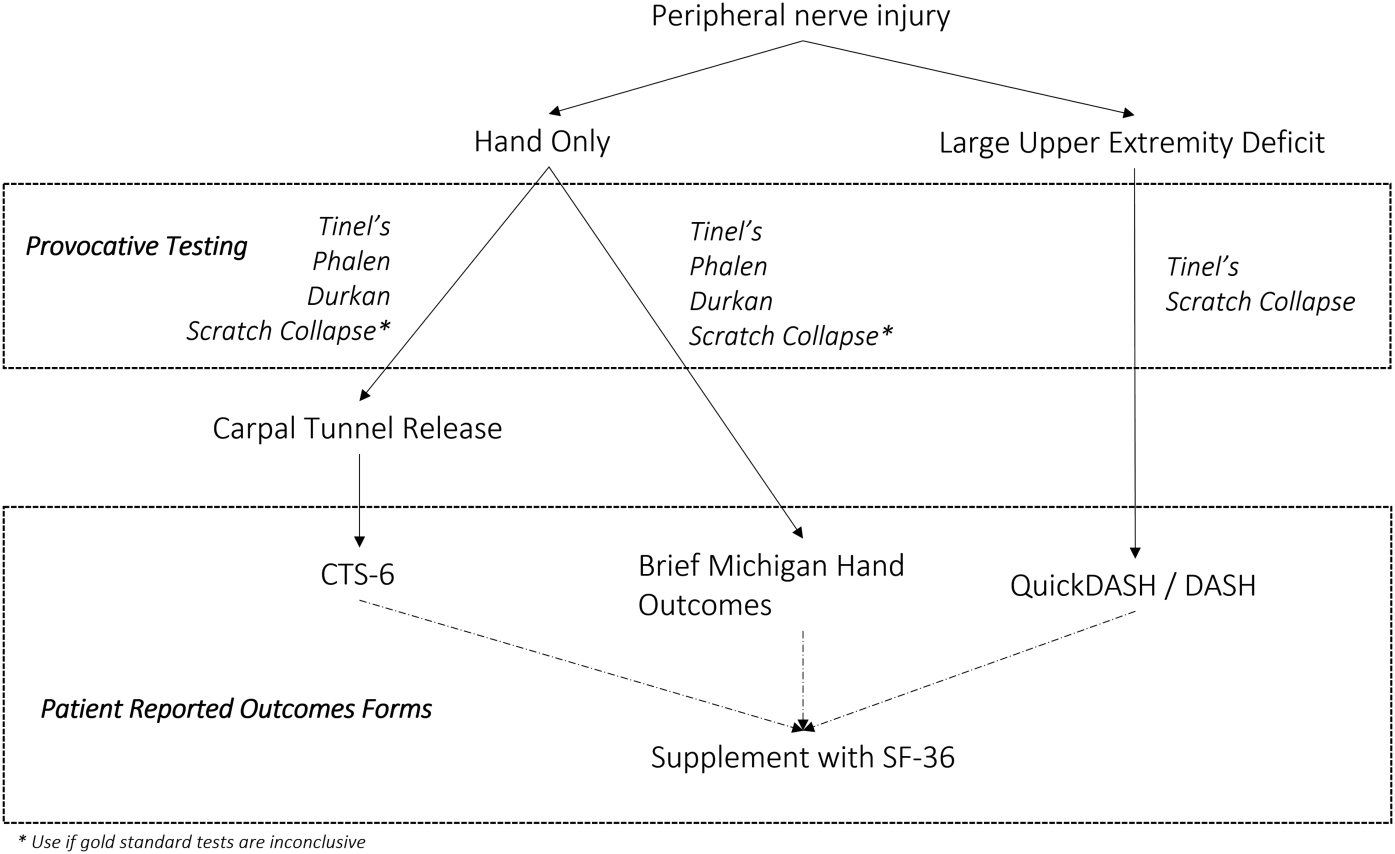
Suggested algorithm for provocative testing and patient-reported outcomes in peripheral nerve injury

There are many measurement tools available to gauge the functional status and progress after PNI and repair, but selecting the optimal test(s) is difficult and lacks standardization across the field. While there is no single test that can give a full clinical picture of a patients' nerve injury and/or recovery, performing every test at each visit is untenable due to practical considerations. Thus, it is important to optimize nerve assessment algorithms to obtain both accurate and relevant data in each unique case ( Fig. 2 ). 14

A PROM can be broad (e.g., SF-36, and DASH to a lesser degree) or specific (e.g. CTS-6). As it pertains to peripheral nerve injuries, the bulk of these assessments focus on the upper extremities. While SF-36 provides a broad view of physical and mental health status, more specific measures are recommended when available. If comparison across various upper extremity injuries and populations is preferred, the QuickDASH is recommended over the DASH, as it is shorter and more practical. For CTS assessment, the CTS-6 is recommended over the BHQ as it also reduces the responder burden. The PSFS can provide a broad understanding of a patient's functional disability following nerve injury, while the Rosen and Lundborg scale can give a more detailed picture of a patient's disability.

Responder burden, as well as resource and time limitations, must be considered when choosing between assessments. The abbreviated versions of many validated assessments such as the DASH, MHQ, and CTS, do not lose reliability or increase error and, thus, are sufficient and recommended. Much of nerve injury recovery is also affected by psychological factors and further studies are needed to determine the extent to which these factors are inhibiting successful recovery. Further study into the effectiveness of tracking HRQoL is also recommended.

While myriad provocation tests (such as tethered median nerve stress, lumbrical provocation, hand elevation, and scratch collapse) have been described in the literature, 23 the Tinel's, Phalen's, and Durkan's tests have endured as the current gold standards for provocative testing. 25

## Limitations

We acknowledge that the discussion of the various provocative testing and patient-reported outcomes are not exhaustive and are limited by our inclusion criteria. For example, discussion of thoracic outlet syndrome and compression neuropathies of lower extremities were not included in this study but offer an avenue for future research. Additionally, further study into international methods, much as Wouters and Colleagues have done, using a similar approach as is demonstrated in this review is an opportunity for future research. 62

## Conclusion

This review provides a guideline for optimizing the battery of provocative tests and patient-reported outcome measures used by surgeons to monitor nerve function before and after peripheral nerve surgeries. As management of PNI continues to improve and becomes increasingly evidence-based, identifying the most appropriate measures of success is imperative for accurately tracking and improving patient outcomes.
